# Molecular Tumor Boards: The Next Step towards Precision Therapy in Cancer Care

**DOI:** 10.3390/hematolrep15020025

**Published:** 2023-04-04

**Authors:** Angela Liu, Paige Vicenzi, Ishna Sharma, Kaci Orr, Christa Teller, Micha Koentz, Heidi Trinkman, Kelly Vallance, Anish Ray

**Affiliations:** 1 Texas College of Osteopathic Medicine, University of North Texas Health Science Center, Fort Worth, TX 76107, USA; angelaliu@my.unthsc.edu (A.L.);; 2 Department of Pediatrics, Dell Children’s Medical Center, Austin, TX 78723, USA; 3 Texas A&M Health Science Center School of Medicine, Bryan, TX 77807, USA; 4 Department of Pediatric Hematology/Oncology, Cook Children’s Medical Center, Fort Worth, TX 76104, USA; 5 Department of Pharmacy, Cook Children’s Medical Center, Fort Worth, TX 76104, USA

**Keywords:** molecular tumor board, precision oncology, genetic profiling, targeted therapy, pediatric oncology

## Abstract

The application of molecular tumor profiles in clinical decision making remains a challenge. To aid in the interpretation of complex biomarkers, molecular tumor boards (MTBs) have been established worldwide. In the present study, we show that a multidisciplinary approach is essential to the success of MTBs. Our MTB, consisting of pediatric oncologists, pathologists, and pharmacists, evaluated 115 cases diagnosed between March 2016 and September 2021. If targetable mutations were identified, pharmacists aided in the evaluation of treatment options based on drug accessibility. Treatable genetic alterations detected through molecular testing most frequently involved the cell cycle. For 85% of the cases evaluated, our MTB provided treatment recommendations based on the patient’s history and results of molecular tumor testing. Only three patients, however, received MTB-recommended targeted therapy, and only one of these patients demonstrated an improved clinical outcome. For the remaining patients, MTB-recommended treatment often was not administered because molecular tumor profiling was not performed until late in the disease course. For the three patients who did receive MTB-recommended therapy, such treatment was not administered until months after diagnosis due to physician preference. Thus, the education of healthcare providers regarding the benefits of targeted therapy may increase acceptance of these novel agents and subsequently improve patient survival.

## 1. Introduction

Pediatric oncology has witnessed a rise in the development and use of targeted therapies, a trend which predictably aligns with the advancements and improved accessibility of whole-genome sequencing. Correlative studies linking genomic variants to clinical significance in malignancies are the foundation of “precision oncology” and have driven the expansion of precision clinical trials [1]. This movement is supported by mounting evidence demonstrating the favorable clinical impact of genetically-informed, or “matched,” therapy [2]. For instance, in a systematic review of fourteen studies including 3328 cancer patients, the progression-free survival was significantly improved in the group receiving MTB-recommended therapy compared to those receiving conventional therapy [3]. Similarly, a case-control study of patients diagnosed with non- small cell lung cancer showed that lack of MTB review was significantly associated with poorer survival outcomes [4]. It remains challenging, however, to interpret complex molecular tumor profiles and apply that knowledge in clinical decision making because the correct interpretation of such complex reports requires expertise from multiple branches of medicine. In response to this rapidly expanding biomarker landscape, an increasing number of institutions across the country are implementing molecular tumor boards (MTBs).

MTBs draw on expertise across various disciplines to translate genetic profiles into actualized precision oncology. These multidisciplinary teams commonly include medical and radiation oncologists, pathologists, geneticists, bioinformaticians, and molecular biologists [5]. However, no standardized protocol exists for MTBs regarding composition, objectives, tools, workflow, or outcome requisites, with the scarce literature available being limited to single-institutional experiences [6]. Other common drawbacks to this approach include a lack of large prospective studies and siloed knowledge gained from the individual practice of precision medicine [7]. Here, we aim to provide a convincing argument and working framework for a multidisciplinary approach toward MTBs.

## 2. Materials and Methods

### 2.1. Our Interdisciplinary Approach to MTB Meetings

**Figure 1:**
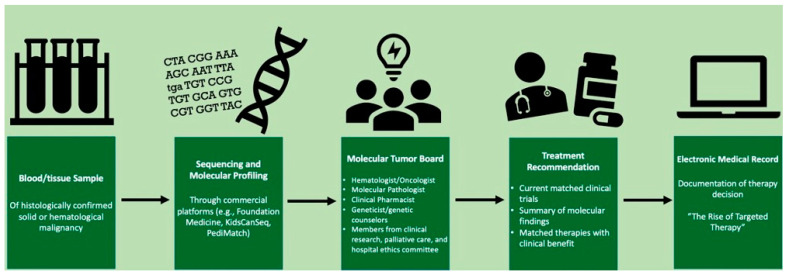
Overview of workflow for tumor genomic profiling and molecular tumor board (MTB) case evaluation.

Our MTB was established in 2019 at the initiative of our pediatric oncology team, with a goal to deliver greater awareness, edification, and applicable skill in precision oncology. Further, the MTB aimed to create access to alternative, cutting-edge treatment options for our patients. Figure 1 shows the workflow for genetic profiling and MTB discussions and recommendations based on these molecular findings. Meetings occur monthly and were adapted to a virtual platform at the onset of the coronavirus disease 2019 pandemic. Any and all provider types at our institution, including medical students, are invited to participate, but core members consist of pediatric oncologists, pathologists, geneticists, nurse coordinators, and clinical pharmacists. To further build the program and prevent knowledge siloing, other specialists who are not members of the patient’s care team, including those external to our institution, have been encouraged to attend and lend their experience. Cases are selected at the recommendation of any provider; typically, however, these include patients who have relapsed or treatment-refractory malignancies. Clinical data (e.g., history of present illness, treatment complications, imaging, and malignancy overview) as well as next-generation sequencing (NGS) panel results and related treatment recommendations are discussed.

At our institution, genomic profiling has been accomplished via Foundation One Medicine, Inc. to detect four main classes of genomic alterations: DNA and RNA genes implicated in solid tumors and/or hematologic cancers via NGS, microsatellite status (MS) via NGS, tumor mutation burden (TMB) via NGS, and programmed death ligand-1 (PDL-1) expression via immunohistochemistry. If targetable mutations are present, clinical pharmacists lead in weighing treatment options and exploring the logistics and feasibility of drug access. The targeted therapies recommended by the MTB are stratified according to three different levels of evidence: Level 1 recommendations have established clinical utility, targeting mutations that have been established in the literature as diagnostic, prognostic, and/or predictive of management in the specific tumor type tested, with an example including the *ALK* p.F1174L mutation in neuroblastoma [8]. Level 2 recommendations have potential clinical utility, as the mutations identified are components of targetable pathways, gene families, and/or functional groups, regardless of the tumor type tested, with an example including the *TSC2* frameshift mutation in osteosarcoma [5]. Level 3 recommendations have limited utility or are being currently investigated, as the mutations identified are not considered targetable at present, with an example including the *MED12* p.G44D mutation in Wilms tumor [8]. The final decision for treatment, including whether to incorporate the recommendations of the MTB, is made at the discretion of the primary oncologist in partnership with the patients and their families.

### 2.2. Role of Clinical Pharmacists

The inclusion of pharmacists in our institutional MTB added a unique skill set to the multidisciplinary team by providing a medication-driven perspective in approaching the genetic findings and suggesting targeted therapies as applicable in a pediatric setting. They also contributed via their assessments of mechanisms, interactions, side effects, dosage forms, availability, and cost. Pharmacists also assist in literature searches, assessing investigational options, and facilitating discussions with pharmaceutical companies on unpublished data necessary for drug administration.

In particular, if the drug was not available commercially, meaning that it was not approved by the Food and Drug Administration (FDA) or the patient’s insurance denied coverage, obtaining the drug via an investigational route became essential. In our experience, these medications were obtained via one of two mechanisms: an established clinical trial or expanded access through the FDA. If a trial was open, enrolling, and the patient qualified, we opened the trial at our site. Otherwise, an attempt was made to utilize the expanded access process established by the FDA. The pharmacist communicated with the drug company to obtain permission for the patient to receive the drug. The company allowed it if no clinical trials were available, and they believed the patient could benefit. If approval was granted, the request then went to the FDA, which weighed the risk versus the benefits for the patient and then decided on approval. If the FDA approved, our institution’s institutional review board also had to approve the protocol for use in the patient. Once all approvals had been received, the drug was ordered and administered. This route was both financially beneficial, as the drug was being provided at no cost, and opened up the potential for therapies that would otherwise not have been an option.

## 3. Results

### 3.1. Financial Impact

The expertise of our clinical pharmacists, an essential component of our multidisciplinary approach, allowed significant cost avoidance in the management of each patient, achieved via single-use, FDA-approved targeted therapy. Financial burden is removed not only from our institution, but also our patients, while informing the use and outcomes of these new yet promising indications. Table 1 lists the cost savings for single-use drugs approved by the FDA. The formation of the MTB has helped overcome some of the hurdles that were faced by individual oncologists attempting to decipher complicated molecular medicine-based reports and incorporating those recommendations into clinical practice.

### 3.2. Disease Settings Evaluated by Molecular Tumor Board

**Figure 2:**
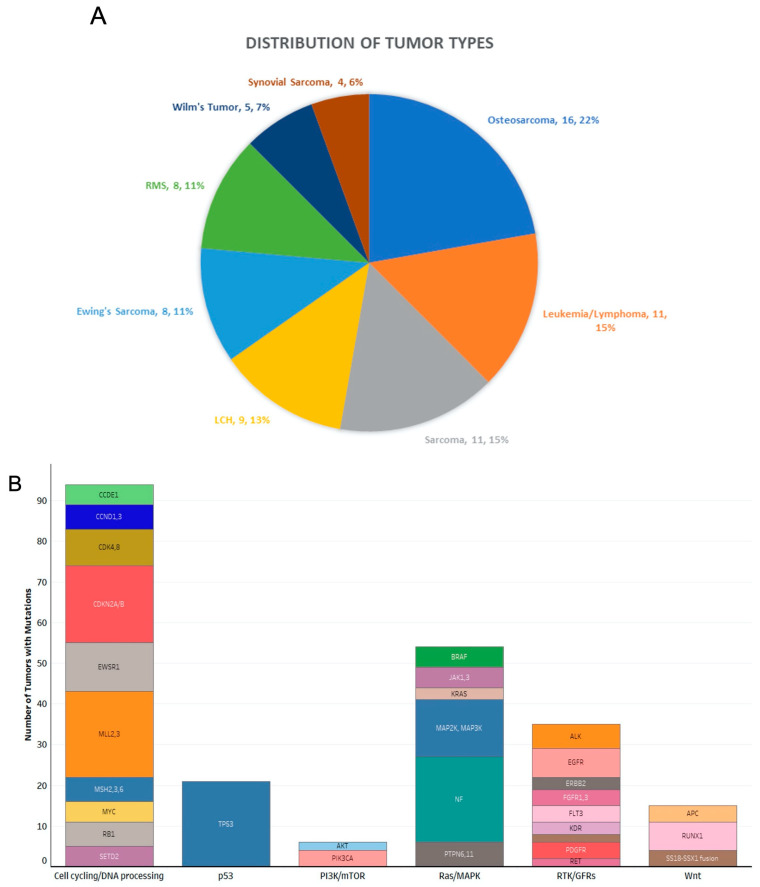
Tumor types and mutated genes evaluated by our MTB. (**A**) Distribution of tumor types among 115 cases. Abbreviations: RMS, rhabdomyosarcoma; LCH, Langerhans cell histiocytosis. (**B**) Incidence of molecular alterations by gene. Genes were grouped into pathways as in [7], and frequencies of alterations were calculated. Abbreviations: GFR, growth factor receptor; MAPK, mitogen-activated protein kinase; mTOR, mammalian target of rapamycin; PI3K, phosphatidylinositol 3-kinase; RTK, receptor tyrosine kinase.

The MTB evaluated 115 cases diagnosed between March 2016 and September 2021. The most common types of cancer evaluated by the MTB included osteosarcoma, leukemia/lymphoma, and sarcomas other than osteosarcoma (Figure 2A). Supplementary Table S1 outlines the type of malignancy identified in each patient and the results of genomic testing, including PD-L1 expression percentage, microsatellite instability level, tumor mutational burden, genomic findings, variants of unknown significance, and recommended immunotherapies and/or clinical trials. Furthermore, this table includes the traditional therapies the patient received and whether they received any targeted therapies recommended by the MTB.

Using Foundation One Medicine testing, Figure 2B shows 223 alterations that were detected across 41 genes grouped into 6 functional pathways, as outlined by Balko et al. [9]. Targetable alterations were found in 85% of genetic mutations (189 out of 223), most frequently occurring in the cell cycle/DNA processing pathway. The most common treatable alterations were those of the genes *TP53* (n = 21; 11%), *MLL* (n = 21; 11%), and *CDKN2A/B* (n = 19; 10%). Many of the types of malignancy evaluated by the MTB only had one patient (i.e., diffuse large B cell lymphoma, medulloblastoma, etc.), or no common genomic findings were found among multiple patients with the same type of malignancy (i.e., acute myeloid leukemia, Wilms tumor, etc.). Of note, out of the sixteen patients with osteosarcoma, 43.8% (7/16) exhibited a *TP53* mutation, 43.8% (7/16) exhibited a *C17orf39* mutation, 31.3% (5/16) exhibited a *CCNE1* mutation, and 25% (4/16) exhibited an *RB1* mutation. Other mutations were shared by patients, including, but not limited to, mutations in *CCND3* (25% or 4/16), *AURKA/B* (25% or 4/16), *CDK4* (18.8% or 3/16), and *GNAS* (18.8% or 3/16). Among nine patients with Ewing’s sarcoma, 88.9% (8/9) exhibited a *EWSR1-FLI1* fusion. Out of nine patients with Langerhans cell histiocytosis, 44.4% (4/9) exhibited a *MAP2K1* mutation, and 44.4% (4/9) exhibited a *BRAF* mutation.

The therapy recommendations for the treatable alterations were classified as follows: 98 (52%) level 1 recommendations, 49 (26%) level 2 recommendations, and 42 (22%) level 3 recommendations. Overall, 3 of 82 patients received targeted therapy based on their identified alterations (“matched”). Of the 34 cases without treatable alterations, 6 were due to a lack of detectable genetic mutations, 9 were due to sample failure, and the remaining 25 patients had detectable but non-treatable genetic alterations.

### 3.3. Impact of Molecular Tumor Board on Treatment Decisions

**Figure 3:**
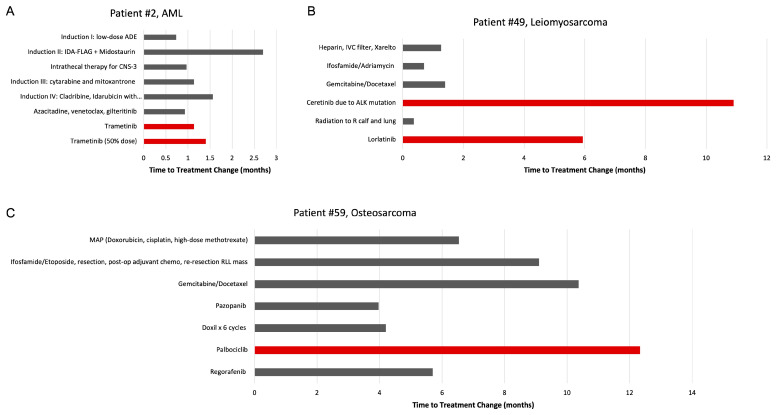
Treatment history of the three patients whose management included therapies recommended by the MTB. Treatments used are shown on the y-axis, and times to change in treatment are displayed along the x-axis. Red columns indicate MTB-recommended therapy. (**A**) Patient 2 with metastatic AML. (**B**) Patient 49 with leiomyosarcoma. (**C**) Patient 59 with metastatic osteosarcoma. Abbreviations: AML, acute myelogenous leukemia; IVC, inferior vena cava; ALK, anaplastic lymphoma kinase.

Among the 115 patients with previously treated disease and for whom the MTB recommended targeted treatment, three were subsequently treated with a MTB-recommended therapy (Patients 2, 49, and 59). Here, we highlight the treatment histories of these three cases in which the recommendations of the MTB influenced management (Figure 3). Although the cases are few in number, they illustrate how the utilization of targeted therapies recommended by a multidisciplinary MTB creates new avenues for the treatment of refractory or relapsed disease.

Patient 2 was an 18-year-old male diagnosed with CNS3 acute myeloid leukemia (AML). Per an AML treatment protocol, the patient received induction chemotherapy but was noted to have 54% blasts at the end of the induction phase with low-dose ADE (cytarabine, daunorubicin, and etoposide) followed by IDA-FLAG (idarubicin, fludarabine, cytarabine, and granulocyte colony-stimulating factor). The patient also received weekly intrathecal triple therapy for CNS involvement. Genomic profiling revealed *RUNX1-FPL22* and *RUNX1-MECOM* gene fusions, an *FLT3* mutation, a mitogen-activated extracellular signal-related kinase (*MEK*) mutation, and several other mutations of unknown clinical significance, including *KRAS, NRAS, ATRX, BLM,* and *WT1* variants. Given his *FLT-3* status, midostaurin, a kinase inhibitor approved by the FDA to treat *FLT-3* mutation-positive AML, was added on day 10 of the second induction attempt, administered for the next 14 days, and repeated on days 8–21 of each cycle.

After a minimal residual disease (MRD) analysis via flow cytometry revealed 9.4% abnormal myeloblasts, a third induction attempt was initiated with cytarabine (days 1–4) and mitoxantrone (days 3–5). Due to persistent residual disease, a fourth induction attempt was initiated several weeks later consisting of cladribine ×5 days, idarubicin with dexrazoxane ×3 days, cytarabine ×3 days, venetoclax ×7 days, and gilteritinib ×14 days (substituting for midostaurin). After the fourth induction attempt, MRD was determined as 7%, at which point the lack of expectation of cure was explained, and the patient understood the need to shift to supportive care. The following month, the patient received individualized therapy with azacitidine dosed at 75 mg/m^2^ on days 1-5, venetoclax at a dose of 100 mg on days 1–14, and gilteritinib at a dose of 120 mg on days 1–28. Administration of venetoclax was ceased early on day 8 at the patient’s request due to concerns of prolonged myelosuppression.

Based on the genomic profiling results, the MTB recommended therapy with binimetinib, cobimetinib, and/or trametinib, all of which are *MEK* inhibitors. Thus, upon the recommendation of the MTB, the patient began therapy with trametinib; however, this was stopped after a month due to signs of rhabdomyolysis. Due to a drastic blast count increase from 3% to 24%, trametinib was ultimately restarted at 50% of the original dose. The patient passed away three months later, one year after initial diagnosis.

Patient 49 was an 18-year-old female diagnosed with leiomyosarcoma. She initially presented with a mass of the right calf and significant hypoxia and chest pain. Biopsy of the calf mass demonstrated leiomyosarcoma. The patient’s respiratory symptoms prompted imaging, which revealed lung lesions that were positron emission tomography (PET)-avid, consistent with tumor thrombi. The patient was extensively anticoagulated with heparin, rivaroxaban, and placement of an inferior vena cava filter and received one cycle of ifosfamide and adriamycin. During this cycle, she experienced complications of right heart strain requiring aggressive management with nitrous oxide, milrinone, and epinephrine. One month later, the patient was started on gemcitabine and docetaxel. She initially demonstrated clinical improvement but then experienced progression of disease after two cycles.

Genomic profiling revealed a *PPFIBO1-ALK* gene fusion, a CD36 N53fs*24 mutation, an anaplastic lymphoma kinase (*ALK*) mutation, and loss of *CDKN2A/B*. Based on these results, the MTB recommended therapy with entrectinib, a *ROS1* and *NTRK* inhibitor, alectinib, an *ALK* inhibitor, brigatinib, an *ALK* and *EGFR* inhibitor, ceritinib, an *ALK* inhibitor, crizotinib, an *ALK* inhibitor, and/or lorlatinib, an *ALK* and *ROS1* inhibitor. Upon the recommendation of the MTB, the patient began therapy with ceritinib. Simultaneously, she also received radiation therapy (45 Gy) to the right proximal calf, resulting in shrinkage of the lower extremity mass. Improvement in the size and PET-avidity of the lung metastases was also observed. Three months later, the calf mass was surgically resected with positive margins. She then received additional radiation therapy (15 Gy) directed at the lungs and the tumor bed in the area of positive margin on the right calf (an additional 18 Gy). Four months later, evidence of progressive disease was identified on imaging and confirmed via thoracoscopic biopsy. For her given apparent resistance to ceretinib, the patient was switched to lorlatinib, a third-generation *ALK* inhibitor, at a dose of 100 mg daily. Lorlatinib was eventually held due to complications of grade 1–2 edema. The patient passed away six months later, nearly two years after initial diagnosis.

Patient 59 was a 25-year-old female diagnosed with osteosarcoma of the left femur at age 12. She was initially treated with a MAP (doxorubicin, cisplatin, and high-dose methotrexate) regimen. Six years later, a right intrathoracic metastasis was identified. The intrathoracic metastasis was initially managed with neoadjuvant ifosfamide and etoposide, resection with positive margins, and two cycles of adjuvant chemotherapy.

While awaiting the initiation of radiation therapy, tumor regrowth was identified, requiring resection of a right lower lobe mass demonstrating parenchymal and pleural involvement with positive margins, which was followed with adjuvant radiation therapy. One year later, recurrence at the same site was identified and managed with gemcitabine and docetaxel; the patient was six months pregnant at the time. Complications of this treatment course included the development of peripheral edema and a pleural effusion causing significant dyspnea, both of which resolved with a pleurocentesis. Two months later, a progressive tumor in the right pleural base and dome of the liver was detected. The patient was started on pazopanib, a *VEGFR* inhibitor, as salvage therapy, which was later stopped entirely due to gastrointestinal toxicity. The patient also received six cycles of doxorubicin with an initial positive response, followed by Yttrium-90 radioembolization of both hepatic lobes.

Genomic profiling revealed mutations in *CDK4, RICTO, C17orf39, CCND3,* and *CDKN2A*, loss of *DAXX*, and amplification of *KDM4C*. Based on these results, the MTB recommended therapy with palbociclib and ribociclib, both of which are *CDK4/6* inhibitors. Thus, upon the recommendation of the MTB, the patient began therapy with palbociclib. After a year on palbociclib, she demonstrated increasing symptoms and signs of disease progression on imaging; therefore, therapy with regorafenib, another *VEGFR* inhibitor, was initiated. The patient passed away six months later, twelve years after initial diagnosis.

## 4. Discussion

MTBs are at the forefront of the shift towards the ever-increasing use of personalized medicine. Mounting clinical evidence indicates that genetically-informed (“matched”) anticancer therapy offers improved clinical benefits compared to non-informed (“non-matched”) therapy. The Biomarker-integrated Approaches of Targeted Therapy for Lung Cancer Elimination (BATTLE) trial was the first biomarker-based study, and it examined 255 previously-treated lung cancer patients [10]. In this 8-week analysis, the median survival of patients who received matched therapy (9.6 months) was significantly higher than that of patients who received non-matched therapy (7.5 months), establishing the foundation for an individual approach to cancer therapy [10]. Furthermore, in an analysis performed by the MD Anderson Cancer Center Initiative, cancer patients who received matched therapy compared with non-matched therapy demonstrated a significantly higher overall response rate, longer time-to-treatment failure, and longer overall survival [11]. Similar to the BATTLE trial, the MD Anderson study indicates the potential of individualized, targeted immunotherapy to improve patient outcomes.

### 4.1. Impact of MTB-Recommended Therapy on Patient Survival

Per the American Cancer Society, the five-year survival rate for AML patients younger than age 20 is 68%, compared to 26% for patients aged 20 and older, suggesting that young age is a favorable factor impacting the overall survival of AML patients [12]. Although the first patient discussed, who was diagnosed with CNS3 AML at age 18, passed away within a year of his diagnosis, a significant factor that contributed to the patient’s premature death was the failure of four induction regimens to render the patient MRD-negative. Furthermore, in a study of 68 adolescents and young adults aged 18 to 39 years with metastatic leiomyosarcoma, median overall survival was reported as 15.0 months [13]. In comparison, the second patient described experienced a more favorable survival rate of 22 months after a diagnosis of metastatic leiomyosarcoma. Unlike the other cases described, early administration of a MTB-recommended targeted therapy (ceritinib) was achieved, as this therapy was initiated only three months after diagnosis. In a study that examined the outcomes of pediatric patients with osteosarcoma, 3-year and 5-year overall survival rates were 79% and 71%, respectively [14]. In comparison, the third patient described demonstrated a relatively long survival period of 12 years after diagnosis.

### 4.2. Need for Tumor Re-Biopsy

Despite the numerous potential benefits of precision oncology, there are limitations that must be addressed. Specifically, targeted immunotherapies for recurrent disease may fail in part because the genomic alterations driving the growth of the recurrence may be unique compared to those that were present in the initial tumor. In a study conducted by Johnson et al., the exomes of 23 initial low-grade gliomas and recurrent tumors resected from the same patients were sequenced [15]. In 43% of cases, at least half of the mutations detected in the initial tumor were not present in the recurrent tumor, including driver mutations in *TP53, ATRX, SMARCA4,* and *BRAF*, indicating that recurrent tumors may be seeded by cells of the initial tumor at an early stage of development, and each tumor accumulates its own set of genomic alterations [15].

Within the same study, tumors from 6 of the 10 patients treated with temozolomide (TMZ) followed a distinct evolutionary path to a high-grade glioma [15]. At recurrence, these tumors were hypermutated and carried driver mutations in the *RB* and *AKT-mTOR* pathways that are classically associated with TMZ-induced tumorigenesis [15]. Similarly, there have been many other reports of immunotherapy-induced resistance, such as that observed with the use of inhibitors of *mTOR* and *MEK/RAF* pathways [16,17]. Notably, therapy with *EGFR* inhibitors, such as erlotinib and gefitinib, has been demonstrated to confer drug resistance through the acquisition of a specific mutation, p.T790M variant [18]. Consequently, re-biopsy of a tumor, especially after the administration of other traditional therapies, including chemotherapy and radiation therapy, is necessary to monitor the genetic evolution of the tumor and guide subsequent treatment decisions.

The outcomes of our study and others raise the question of whether primary or recurrent, metastatic lesions are more appropriate for genomic profiling. Unfortunately, in our study, genetic testing was often conducted months after the initial diagnosis. Thus, the MTB was limited to the analysis of genomic alterations identified in relapsed or previously-treated tumors, rather than those of the primary tumor, a shortcoming that we have attempted to overcome by encouraging the early performance of tumor genomic profiling.

For example, in regards to patient 2, the tumor did not undergo genetic testing until 8 months after the initial diagnosis. Thus, the limited efficacy of the recommended therapy (trametinib) could be attributed to this late administration, as the patient had already received four rounds of induction chemotherapy. Similar to patient 2, patient 59 did not undergo tumor genomic profiling and receive targeted therapy until four years after initial diagnosis. Thus, future trials examining the impact of precision oncology may need to re-biopsy the malignancy multiple times throughout a patient’s disease course to ensure that the results of molecular analysis, which are used in clinical decision making, are most representative of the evolving heterogeneity of the tumor.

### 4.3. Matching Therapy to MTB Recommendations

Given that the MTB at our children’s hospital is fairly new, the MTBs at other institutions, such as the University of California San Diego (UCSD) Moores Cancer Center, provided insight into areas of improvement for our MTB. Similarly to our MTB, the physicians at UCSD ultimately made decisions regarding management in conjunction with the patients, even if such decisions did not follow the recommendations of the MTB, which were considered simply advisory [19]. In our MTB and at UCSD, deviation from MTB recommendations likely highlights physician preference to adhere to established, conventionally recognized therapies, including chemotherapy and radiation therapy. Thus, limitations of our MTB that should be addressed in future studies include the small sample size of patients who ultimately received MTB-recommended therapy and the lack of a control group consisting of patients who did not receive MTB review.

### 4.4. Standardization of MTB Recommendations

A study conducted at the Memorial Sloan Kettering Cancer Center (MSKCC) demonstrates the need for standardization of the evidence supporting MTB recommendations [20]. Targeted therapies were recommended for 21 of the 41 cases evaluated in the study [20]. The majority of the targeted therapy recommendations lacked published evidence of the clinical efficacy of targeted therapies [20]. Specifically, the majority of recommendations were based upon either pre-clinical evidence (level 3) or hypothetical rationales based upon biological evidence and inferred molecular mechanisms (level 4) [20]. This is similar to our study, in which almost half of the cases (48%) were designated as level 2 (may have potential clinical utility) or level 3 (have limited utility or are currently being investigated) [8]. The lack of published evidence to support the proposed recommendations hampers the adoption of MTB-recommended therapies. Thus, standardization of the evidence that serves as the foundation of MTB recommendations is crucial to improve the clinical efficacy of such therapies [21].

### 4.5. MTB Format and Turnaround Time

A systematic analysis of 40 studies, including 6,303 cases discussed in MTBs globally, demonstrated that the core members of an MTB should include clinical oncologists (present in 100% of studies) and pathologists (90.6%) [5]. Other common and important MTB members included geneticists (68.7%), bioinformaticians (37.5%), and molecular biologists (25%) [5]. Although less commonly present (21.9%) in these studies [5], clinical pharmacists proved instrumental in securing medications for off-label use and determining the feasibility of drug access as members of our MTB, yielding substantial cost savings both for our institution and for patients. While our MTB did include oncologists, pathologists, and geneticists, we should consider the inclusion of bioinformaticians and molecular biologists, who can lend their expertise as we implement novel NGS techniques and continue to interpret large amounts of molecular data [5]. The addition of a moderator, similar to the role created at UCSD for a senior-level medical oncologist experienced in clinical trials, genomics, and immunotherapy, may help guide and streamline MTB decision making [19]. Additionally, we may consider the inclusion of genetic counselors, who have been shown to provide benefits to MTBs [22,23]. When a germline mutation is detected in a patient, genetic counselors may refer family members of patients to receive early cancer screening, improving clinical outcomes [22,23].

Of these 40 studies, the mean turnaround time from when genomic profiling was requested to when MTB recommendations were provided was 38.4 days, with a range of 12.4 to 86 days [5]. The median turnaround time at our institution (33 days) was comparable to that of these studies [5,24,25]. To ensure that targeted therapy is initiated in a timeframe in which it is clinically beneficial, efforts should be made to reduce both the turnaround time for tumor genomic profiling and for MTB analysis. Thus, a period of 28 days may offer a balance between the need to initiate treatment and the time required for high-quality molecular testing and thorough MTB interpretation, discussion, and decision making [5,26].

## 5. Conclusions

The present study demonstrated the utility of a multidisciplinary approach and highlighted the need to perform genetic profiling and initiate matched therapy immediately after tumor detection. Our primary aim in describing the formation of our MTB and the application of our recommendations to three cases is to serve as a model to other institutions, particularly smaller, non-academic institutions such as ours, who may be considering establishing a MTB. In the future, we hope to publish a more comprehensive review with a larger sample size. Future precision oncology studies should also standardize MTB recommendations to maximize those based on level 1, or published evidence. Precision medicine will predictably become a pillar of standard care in pediatric oncology to meet the growing demand for alternative treatments that surpass traditional chemotoxic agents in potency and side effect profile. In the presence of an increasingly expansive and complex biomarker landscape, there is urgency to establish the interdisciplinary foundation of MTBs.

## Figures and Tables

**Table 1:** Estimated cost and thus savings for single-use drugs approved by the FDA.

Drug	# of Patients Treated	# of Cycles Dispensed	Cost Avoidance (Estimated Medication Cost)
Ceritinib	1	11	USD 132,145
Dabrafenib	1	31	USD 250,898.20
Crizotinib	3	4	USD 84,072
Lorlatinib	2	26	USD 645,568
Alisertib	2	29	N/A
Entrectinib	1	4	USD 29,972
Tazemetostat	1	1	USD 5425
Selumetinib	4	15	USD 286,321
Selinexor	2	6	USD 8726.58
Nirogacestat	2	2	N/A
		Total	USD 1,443,127.70

## Data Availability

The data presented in this study are available on request from the corresponding author.

## Supplementary Materials

The following supporting information can be downloaded at: https://www.mdpi.com/article/10.3390/hematolrep15020025/s1, Table S1: Foundation One Medicine genomic testing results and targeted therapy recommendations for all patients evaluated by the MTB, organized by malignancy type. Abbreviations: AML, acute myeloid leukemia; B-ALL, B Cell Acute Lymphoblastic Leukemia; DLBCL, Diffuse large B-cell lymphoma; DSRCT, Desmoplastic small round cell tumor; HCC, hepatocellular carcinoma; LCH, Langerhans cell histiocytosis; RCC, renal cell carcinoma; RMS, rhabdomyosarcoma; T-ALL, T Cell Acute Lymphoblastic Leukemia.
