# Supplementary material for: Molecular Tumor Boards: The Next Step towards Precision Therapy in Cancer Care
Source: Hematol Rep. 2023 Apr 4;15(2):244–55. doi: 10.3390/hematolrep15020025 (PMC10123678; doi:10.3390/hematolrep15020025)
Supplement: Supplementary file 1 [file hematolrep-15-00025-s001.zip › hematolrep-2176609-supplementary.pdf]

Supplementary Table S1. Foundation One Medicine genomic testing results and targeted therapy recommendations for all patients evaluated by the MTB, organized by malignancy type.

Diagnosis	PD-L1	MS	TMB	Genomic Findings	Recommended Therapies & Trials	Traditional Tx	Targeted Tx	MTB date	VUS
AML	unknown	stable	low (2)	no significant findings;<br>variants of unknown significance only	none			DOD 3/3/2020	BLM<br>ERBB4<br>FHIT<br>FLYWCH1<br>IKBKE<br>MED12<br>MYST3<br>PLCG2<br>PTCH1<br>PTPN11<br>TET2<br>TP53<br>WDR90
AML	unknown	stable	low (0)	KRAS G12S<br>NRAS Q61R<br>ATRX W2139*<br>BLM Y736fs*5<br>RUNX1-FPL22 fusion<br>RUNX1-MECOM fusion<br>WT1 V379fs*71	Binimetinib<br>Cobimetinib<br>Trametinib<br>1 trial	AML16<br>IDA-FLAG<br>Hydroxyurea<br>5/4/2020	Trametinib<br>1/28/2020<br>Gilteritinib<br>2/19/2020<br>d/c 5/4/2020	2/1/2020<br>DOD 6/7/2020	BLM<br>C11ORF30 (EMSY)<br>CHEK2<br>KMT2A (MLL)<br>MYD88<br>NTRK3<br>S1PR2<br>SETD2
AML	unknown	stable	low (2)	CSF3R T618I	Ruxolitinib<br>8 trials				CD22<br>FGF14<br>FOXO3<br>MED12<br>NCOR2<br>RICTOR<br>TSC1<br>TSC2<br>ZND217
AML	unknown	unknown	low (3)	NUP214-ABL1 fusion<br>ATM R2598Q<br>CDKN2A p16IKN4a I49T<br>GATA1 splice site 220+1G>T<br>RUNX1 truncation exon 2	[illegible]<br>Bosutinib<br>Dasatinib<br>Imatinib<br>Nilotinib<br>Ponatinib<br>4 trials<br>ATM:<br>Olaparib<br>Rucaparib<br>Niraparib<br>1 trial				
AML	unknown	stable	unknown	KMT2A (MLL-MLLT6 fusion)<br>KMT2C (MLL3)	10 clinical trials				
AML	unknown	stable	low (1)	BRCA2<br>NPM1<br>KDM6A<br>PTPN11	BRCA2<br>Niraparib<br>Olaparib<br>Rucaparib<br>Talazoparib<br>1 trial<br>NPM1<br>10 trials				
Angiosarcoma	**	**	low (3)	RAF1<br>AXIN1 A526V<br>PDCD1LG2 (PD-L2) R70H<br>PTPN6 H445fs*101<br>TP53 R248W	Trametinib<br>Cobimetinib<br>Sorafenib<br>Regorafenib<br>4 trials				
Anglomyxoma	0%	stable	low (0)	APC loss	none				
B-ALL	0%	**	**	**sample failure**	**				
Chordoma	unknown	unknown	unknown	unable to reach pt for consent for tissue - sample failure on previous attempt					
Chordoma	0%	**	**	**sample failure**	**				
Chordoma	0%	**	**	**sample failure**	**				
Colon Adenocarcinoma	unknown	unknown	unknown	NF1 L2678_V2685>*<br>TP53 P80fs*43<br>MYST3<br>NOTCH2 G929fs*2<br>PRKAR1A V164fs*5	NF1<br>Cetuximab<br>Panitumumab<br>Tremetinib<br>5 trials<br>TP53<br>1 trial				BRCA2<br>CSF1R<br>FGFR1<br>KDM5A<br>MRE11A<br>STK11
DLBCL	**	**	**	**sample failure**	**				
DSRCT	unknown	unknown	unknown	EWSR1-WT1 fusion<br>CD36 L360*	4 trials	AEWS1031 EOT 2016			EPHA5<br>GRP124<br>TSC2
DSRCT	unknown	unknown	low (3)	AKT3<br>EWSR1-WT1 fusion	Everolimus<br>Temsirolimus<br>8 trials	Salvage Chemo - Temozolomide/ Irinotecan		DOD<br>2/15/2017	CREBBP<br>CTNNA1<br>DNM2<br>FAF1<br>FAM123B<br>MLL3<br>MSH3<br>NOTCH2<br>NUP98<br>SYK<br>TCF3<br>WT1
DSRCT	unknown	unknown	unknown	EWSR1-WT1 fusion<br>PRKAR1A loss	4 trials				

Diagnosis	PD-L1	MS	TMB	Genomic Findings	Recommended Therapies & Trials	Traditional Tx	Targeted Tx	MTB date	VUS
Ewing's Sarcoma	unknown	unknown	unknown	EWSR1-FLI1 fusion	Everolimus<br>Temsirolimus<br>5 trials			DOD<br>8/13/2016	
Ewing's Sarcoma	0%	stable	low (2)	EWSR1-FLI1 fusion<br>TP63 T566M	4 trials	AEWS0031 EOT<br>12/2019			CBL<br>EGFR<br>FANCL<br>HGF<br>KDM2B<br>LRP1B<br>MAP3K7<br>PDCD11<br>PLCG2<br>PTCH1<br>SETD2<br>SPEN
Ewing's Sarcoma	unknown	stable	low (2)	RB1 loss	none	VCR/Doxo/CTX<br>EOT 11/18/2019			CREBBP<br>DAXX<br>EPHA3<br>EPHB1<br>IGF1R<br>IRF8<br>KMT2C (MLL3)<br>MAF<br>MYO18A<br>NUP98<br>PTCH1<br>SETD2<br>SGK1
Ewing's Sarcoma	0%	stable	low (4)	CCND1<br>EWSR1-FLI1 fusion (type 10/5)<br>CDKN2A/B p16INK4a loss and p14ARF loss exons 2-3	Abemaciclib<br>Palbociclib<br>Ribociclib<br>11 trials				BCORL1<br>CPS1<br>HIST1H1C<br>KMT2A (MLL)<br>MAP3K1<br>MED12<br>MLH1<br>NCOR2<br>PTPN6<br>SETBP1<br>TSC1<br>TYK2
Ewing's Sarcoma	unknown	stable	low (4)	CCND1<br>CDK4<br>EWSR1-FLI1 fusion (type 8/6)<br>CKS1B<br>EP300 P2310fs*44<br>MCL1<br>PDCD1	CCND1<br>Abemaciclib<br>Palbociclib<br>Ribociclib<br>10 trials<br>CDK4<br>Palbociclib<br>Ribociclib<br>10 trials<br>EWSR1<br>7 trials				APH1A<br>AR<br>ARID1A<br>BCL11B<br>BLM<br>BRIP1<br>DOT1L<br>FGFR3<br>HSP90AA1<br>PLCG2<br>PTPN11<br>PTPN6<br>STAT6<br>YY1AP1<br>XNF703
Ewing's Sarcoma	0%	stable	low (0)	EWSR1-FLI1 fusion<br>CDKN2A/B loss<br>MYST3	4 trials				CHEK2<br>FLYWCH1<br>IRS2<br>NOD1<br>PDGFRB<br>RELN<br>TSC1<br>ZNF703
Ewing's Sarcoma	1%	stable	low (0)	EWSR1 (FLI1 fusion; type 2)	5 trials				CRKL<br>EWSR1<br>KMT2C<br>PDGFRA I543T<br>SPEN<br>TSC1
Ewings, Recurrent	1%	stable	low (0)	EWSR1	4 trials			12/1/2020	ATM<br>ERG<br>MLL2<br>RELN<br>RUNX1
Ewings, Recurrent	0%	stable	low (0)	EWSR1 (FLI1 fusion)<br>CDKN2A/B loss<br>(Same results w/KCS)	3 clinical trials				PRDM1<br>PRKDC<br>RNF43
Fibrosarcoma	unknown	unknown	unknown	ETV6 ETV6-NTRK3 fusion<br>DDKN2A p16INK4a R58* and p14ARF P721L	6 trials				
Germ Cell Tumor	unknown	unknown	unknown	KRAS G13D<br>TP53 R249S	Trametinib<br>Cobimetinib<br>6 trials				
Glioma (brain)	unknown	stable	unknown	HGF<br>TCL1A<br>TP53	5 trials				
Glomorus Tumor	1%	stable	low (2)	PDGFRB R561_E563>Q	Sunitinib<br>3 trials			7/1/2021	EPHA3<br>ERBB2<br>PCLO<br>PDCD11<br>SETD2<br>TSC1<br>FLYWCH1<br>ROS1<br>HNF1A<br>SETBP1
Granular Cell Tumor	0%	stable	low (2)	none	none				

Diagnosis	PD-L1	MS	TMB	Genomic Findings	Recommended Therapies & Trials	Traditional Tx	Targeted Tx	MTB date	VUS
HCC - Fibromellar	unknown	stable	low (2)	CDKN1B A121fs*24<br>JAK3	none				ARID1A<br>ATR<br>CIC<br>JAK3<br>RPTOR<br>SPTA1<br>ZNF217
Hepatoblastoma	0%	stable	low (1)	CTNNB1 D32, S29F	10 trials	AHEP1531<br>EOT 3/1/3031			AXL<br>CREBBP<br>LRP1B<br>LRRK2<br>MAP3k1<br>MTOR<br>PDGFRA D444H
Hepatoblastoma	0%	stable	low (2)	CTNNB1 G34V<br>TP53 N263D	10 trials			1/1/2020	
Histiocytic Malignancy	20%	stable	low (4)	SETD2	none	Clofarabine - 2 cycles	Nivolumab<br>12/14/2020<br>Nivo + Ipibulumab<br>2/18/2021	Aug 2020,<br>Oct 2020<br>March 2021<br>TCH Rare Tumor Board May 2021	BRIP1<br>DOT1L<br>EGFR<br>HDAC4<br>MCL1<br>MLL2<br>SMARCA1<br>WHSC1 (MMSET)
Hodgkins Lymphoma	unknown	unknown	unknown	None	none				
Inflammatory Myofibroblastic Tumor	50%	stable	low (1)	ALK TPM4-ALK fusion	Brigatinib<br>Ceritinib<br>Crizotinib<br>Alectinib<br>Lorlatinib<br>10 trials			6/1/2021	CUX1<br>IRS2<br>KDM6A<br>PRKDC<br>RNF43<br>SUZ12
LCH	unknown	unknown	unknown	MAP2K1<br>TBL1XR1	MAP2K1<br>Trametinib<br>Selumetinib<br>2 trials	Ara-C<br>Hydrocortisone			AXIN1<br>CIC<br>FBXW7<br>GNAS<br>KMT2C (MLL3)<br>MY018A<br>TSC1
LCH	unknown	**	**	**sample failure**	**	LCH0115			
LCH	unknown	**	**	BRAF R506_K507insLLR	Trametinib<br>Selumetinib	TXCH LCH0115<br>Cytarabine Arm			ARID1A<br>CIC<br>ECT2L<br>FBX011<br>MAP3K1<br>MLL2<br>MYO18A<br>NKX2-1<br>SYK<br>TSC1
LCH	failed	failed	failed	BRAF	Binimetinib<br>Cobimetinib<br>Regorafenib<br>Selumetinib<br>Sorafenib<br>Trametinib<br>2 trials			11/1/2020	
LCH	unknown	stable	low (0)	BRAF	Binimetinib<br>Cobimetinib<br>Dabrafenib<br>Encorafenib<br>Regorafenib<br>Selumetinib<br>Trametinib<br>Vemurafenib<br>3 trials			11/1/2020	
LCH	unknown	unknown	unknown	MAP2K1 (KCS only results)					
LCH	unknown	stable	low (0)	BRAF V600E	Dabrafenib<br>Dabrafenib + Trametinib<br>Encorafenib + Binimetinib<br>Regorafenib<br>Selumetinib<br>Trametinib<br>Vemurafenib<br>Vermurafenib + Cobimetinib				C11ORF30 (EMSY)<br>MLL2<br>SPEN
LCH	unknown	stable	low (0)	MAP2K1 (MEK1) Q56P	Binimetinib<br>Cobimetinib<br>Trametinib<br>3 trials			3/1/2020	
LCH	unknown	stable	low (1)	MAP2K1 (MEK1)<br>PASK	MAP2K1 (MEK1)<br>Binimetinib<br>Cobimetinib<br>Selumetinib<br>Trametinib<br>2 trials				APC<br>CRLF2<br>P2RY8

Diagnosis	PD-L1	MS	TMB	Genomic Findings	Recommended Therapies & Trials	Traditional Tx	Targeted Tx	MTB date	VUS
Leiomyosarcoma	0%	stable	low (2)	PPFIBO1-ALK fusion<br>CD36 N53fs*24<br>CDKN2A/B loss	Entrectinib<br>Alectinib<br>Brigatinib<br>Ceritinib<br>Crizotinib<br>Lorlatinib<br>10 trials	Gemcitabine & Taxotere (progressed after 2 cycles)	Ceritinib 3/2019; progression 2/2020; Lorlatinib 3/2020	6/1/2019, DOD 10/19/2020	
Leiomyosarcoma	5%	stable	low (3)	PPFIBP1-ALK fusion<br>CD36 N53fs*24<br>CDKN2A/B p16INK4a R80* and p14ARF P94L - subclonal loss	Alectinib<br>Brigatinib<br>Ceritinib<br>Crizotinib<br>Lorlatinib<br>10 trials				
Lung Adenocarcinoma	20%	stable	low (1)	ROS1 SDC4-ROS1 fusion	Brigatinib<br>Ceritinib<br>Crizotinib<br>Entrectinib<br>Lorlatinib<br>10 trials	benign			ATRX<br>AXL<br>DDR2<br>EPHB4<br>PPP2R1A<br>PTCH1<br>RAD54L
Lymphoblastic Leukemia/ Lymphoma	unknown	stable	low (2)	CDKN2A/B CDKNEA loss exon 1<br>CDKN2B loss exon 2<br>MTOR S2215Y<br>NT5C2 R367Q<br>PAX5-PTPLAD2 fusion	none				
Lymphoma, lymph node anaplastic large cell	unknown	unknown	low (2)	NPM1-ALK fusion<br>PIM1 amplification<br>STAG2 rearrangement exon 5	Alectinib<br>Brigatinib<br>Ceritinib<br>Crizotinib<br>5 trials				
Malignant Neoplasm	unknown	unknown	low (1)	none	none	ARST0531			BRCA2<br>CRLF2<br>ECT2L<br>FAF1<br>MIB1<br>MSH3<br>NCOR2
Malignant Peripheral Nerve Sheath Tumor	0%	stable	intermediate (7)	NF1 splice site 4431-1G>A<br>CDKN2A/B loss<br>SUZ12 R196fs*15<br>TP53 R280K	Cobimetinib<br>Trametinib<br>9 trials				ATM<br>CTNNB1<br>DNM2<br>MAP3K6<br>ETV1<br>MSH2<br>MSH6<br>NFE2L2<br>NTRK1<br>RICTOR<br>ROS1
Medulloblastoma	unknown	stable	low (0)	JAK1 R724L<br>MLL2 E1207fs*4	none				FBXO11<br>GTSE1<br>LMO1<br>MYCN
Myofibroblastic Tumor	0%	unknown	unknown	ALK THBS1-ALK fusion	Alectinib<br>Brigatinib<br>Ceritinib<br>Crizotinib<br>Lorlatinib<br>Entrectinib<br>10 trials				
Osteosarcoma	0%	stable	unknown	ARAF<br>C17orf39	Sorafenib<br>10 trials	AOST0331 EOT 1/27/2020		7/1/2020	BRCA2<br>CEBPA<br>DDX3X<br>EGFR<br>FLCN<br>KDM6A<br>MAGED1<br>MAP2K4<br>MED12<br>MLL2<br>NOTCH2<br>RPTOR<br>SMC3
Osteosarcoma	unknown	unknown	unknown	CDK4<br>RICTO<br>C17orf39<br>CCND3<br>KDM4C amplification<br>CDKN2A p16INK4a I49T<br>DAXX loss	Palbociclib<br>Ribociclib<br>8 trials	Ifos/VP16<br>EOT 2013<br>Pazopanib (salvage) 5/2/2016<br>d/c 8/29/2016<br>Doxil x 6 cycles<br>Regorafenib 8/13/2018	Palbociclib 8/8/2017 x1 yr	DOD 2/8/2019	

Diagnosis	PD-L1	MS	TMB	Genomic Findings	Recommended Therapies & Trials	Traditional Tx	Targeted Tx	MTB date	VUS
Osteosarcoma	1%	stable	low (2)	RET<br>MCL1<br>RAD21<br>TP53	2 trials				ABL1<br>APH1A<br>ATM<br>BIRC3<br>CBL<br>CHEK1<br>EGFR<br>ERG<br>GPR124<br>KMT2A (MLL)<br>LRRK2<br>PRKDC<br>RASGEF1A<br>RET<br>RUNX1<br>SDHD<br>SETBP1<br>U2AF1
Osteosarcoma	unknown	stable	low (2)	CDK8	none				
Osteosarcoma	0%	stable	low (0)	AURKA<br>GNAS<br>LRP1B<br>RAD21<br>ZNF217	2 trials				
Osteosarcoma	unknown	stable	intermediate (6)	CCNE1<br>RB1<br>TP53	none				
Osteosarcoma	low (1%)	unknown	low (1)	C17orf39<br>CCNE1<br>FLT1 S287F<br>RB1 splice site 2450_2489+76del116 - subclonal<br>TP53 rearrangemnt exon 2	none				
Osteosarcoma	0%	stable	unknown	MYC duplication exons 2-3<br>C17orf39<br>CCNE1 amplification - equivocal<br>MAP2K4 rearrangement intron 6<br>RB1 A18fs*3<br>TP53 R248Q1	5 trials				
Osteosarcoma	10%	stable	intermediate (12)	KDR<br>KIT<br>PDGFRA<br>AURKB<br>CCND3<br>TP53 R290L - subclonal	KDR:<br>Sunitinib<br>3 trials<br>KIT:<br>Imatinib<br>Nilotinib<br>Sorafenib<br>Sunitinib<br>8 trials<br>PDGFRA:<br>Imatinib<br>3 trials				
Osteosarcoma	0%	stable	low (3)	SUFU A25fs*23<br>DNM2 rearrangement intron 19	4 trials				
Osteosarcoma	0%	**	**	**sample failure**	**				
Osteosarcoma	0%	stable	low (3)	AKT2<br>AURKA<br>AXL<br>MYC<br>RICTOR<br>ARFRP1<br>C17orf39<br>CCNE1<br>CRKL<br>FGF10<br>GNAS<br>TP53 rearrangement intron 6	17 trials				
Osteosarcoma	unknown	unknown	low (3)	CDK4<br>AURKB<br>C17orf39<br>CCND3<br>IGF1R<br>NF2 loss exon 1<br>CDKN2A loss<br>CDKN2B loss exons 1-2<br>FHIT loss exon 5<br>LRP1B loss exons 4-7	CDK4:<br>Palbociclib<br>Ribociclib<br>5 trials<br>NF2:<br>Everolimus<br>Temsirolimus<br>10 trials				

Diagnosis	PD-L1	MS	TMB	Genomic Findings	Recommended Therapies & Trials	Traditional Tx	Targeted Tx	MTB date	VUS
Osteosarcoma	0%	stable	low (5)	KIT<br>PDGFRA<br>BCL2L2<br>KDR<br>RB1 G310fs*8, inversion exon 8<br>TP53 splice site 993+1G>A	KIT<br>Sorafenib<br>Imatinib<br>Nilotinib<br>Sunitinib<br>10 trials<br>PDGFRA<br>Imatinib<br>2 trials				
Osteosarcoma	low (1%)	stable	low (4)	CDK4<br>BCL2L2<br>CCND3<br>GNAS<br>NFKBIA<br>FANCE truncation exon 10	Palbociclib<br>Ribociclib<br>8 trials				
Osteosarcoma	0%	stable	low (3)	C17orf399<br>CCNE1<br>TP52 R342*	none				
Peritoneum Mesothelioma				BCL2 H20Q	2 trials				
Pre B-ALL	0%	stable	low (2)	TP53 V272M		AALL1131<br>Blinatumomab (d/c 5/4/2021)<br>Inatumomab			
RCC	0%	stable	intermediate (7)	PIK3Ca E1037K<br>CDKN2A/B p16INK4a and p14ARF R122Q<br>KRI1-KEAP1 fusion<br>TP53 splice site 415_560-14del213	Everolimus<br>Temsirolimus<br>10 trials			7/1/2019	
RCC, recurrent	0%	stable	low (1)	TFE3<br>CDK2NA/B loss<br>KDM5C loss	TFE3<br>Carbozantinib<br>Sunitinib<br>10 trials			8/1/2020	
Rhabdoid Tumor	0%	stable	low (6)	SMARCB1 loss	Tazemetostat<br>10 trials			6/1/2020	
Rhabdoid Tumor	unknown	unknown	unknown	SMARCB1 truncation intron 2<br>MAP3K7N463S	4 trials			DOD 3/16/2016	BRCA2<br>CDKN2A<br>CREBBP<br>MLL2<br>MYO18A<br>TSC2
RMS	unknown	unknown	low (2)	ASXL1-SBNO2 rearrangement<br>RB1 loss exons 2-27<br>TP53 T329fs*8<br>ZND703	none	ARST0331<br>ARST0921 EOT 10/2018			APC<br>DOT1L<br>MLL2<br>MTOR
RMS	0%	stable	low (4)	CDK4<br>PAX3-FOXO1 fusion	Palbociclib<br>Ribociclib<br>10 trials	RMS13<br>ARST0921		5/1/2020<br>DOD 2/19/2021	ACTB<br>CCT6B<br>CUX1<br>JAK2<br>PBRM1<br>PCLO<br>PRDM1
RMS	low (1%)	stable	low (3)	BCOR P1258fs*36<br>NOTCH1 M1737_Y1738insPPPPAQLHFM	none	RMS13 - stopped 9/2019<br>ARST1431 - 12/2019; NED 3 mon OT			BCORL1<br>GATA2<br>HDAC7<br>KEAP1<br>NOTCH1<br>SYK<br>WDR90
RMS	unknown	unknown	unknown	NF1 S1329*<br>MYC amplification<br>ETV6 truncation exon 5<br>SUZ12 splicesite 917+1G>A<br>TP53 Q167_D184>H	NF1:<br>Cobimetinib<br>Trametinib<br>4 trials<br>MYC:<br>3 trials	ARST0331 (12/2015 - completed)<br>Relapse - ARST0921 (x2 cycles)<br>Progression<br>Salavage chemo 3/2016 (ifos/doxo)<br>ARST0332		DOD 2/28/2017	ATRX<br>AXL<br>CD36<br>FANCL<br>FHIT<br>MYST3<br>P2RY8<br>PAG1<br>PCLO<br>PIK3R1<br>POT1<br>PRKDC<br>RAD21<br>RELN
RMS, Alveolar	0%	stable	low (2)	CDK4<br>PAX3-FOXO1 fusion	10 trials				BLM<br>CD36<br>DOT1L<br>FANCC<br>FANCF<br>FBX011<br>FLT3<br>MTOR<br>PCLO<br>PLCG2<br>SETBP1<br>SPEN<br>STAT6

Diagnosis	PD-L1	MS	TMB	Genomic Findings	Recommended Therapies & Trials	Traditional Tx	Targeted Tx	MTB date	VUS
RMS, Embryonal	0%	stable	low (3)	NF1 loss exons 4-8<br>FGFR1<br>CDKN2A/B loss<br>MYST3<br>RAD21<br>ZNF703	NF1<br>Selumetinib<br>Trametinib<br>16 trials	ARST0331		7/1/2021	AR L254V<br>EBF1G501V<br>GPR124<br>MRE11A D131N<br>PAG1<br>PDGFRA E263A<br>PRKDC L3900V<br>RELN A150V<br>RUNX1T1<br>TNFAIP3T647P<br>TSC2 R1409W<br>TUSC3
RMS, Embryonal	10%	stable	low (2)	MALT1<br>TP53	none			1/1/2021	BCL6<br>CRLF2<br>FBXO11<br>HDAC4<br>IGF1R<br>LRP1B<br>MSH2<br>MSH6<br>PASK<br>SOX10<br>SYK<br>WDR90
RMS, recurrent	0%	stable	low (0)	CDK4<br>MDM2<br>FOXO1 (COL4A4 fusion)<br>FRS2<br>MYCL1<br>PAX3 (FOXO1 fusion)	CDK4:<br>10 trials<br>MDM2:<br>7 trials				BRCA2
Sarcoma	0%	**	**	**sample failure**	**				
Sarcoma	0%	stable	low (3)	BCOR-CCNB3 fusion	none	ARST1321			AR<br>MLH1
Sarcoma	unknown	unknown	unknown	NF2 truncation intron 13<br>PASK R451*	Everolimus<br>Temsirolimus<br>4 trials	ARST (VAC/VI)<br>progression 3/2018	Pembrolizumab<br>3/2018	DOD<br>8/11/2018	
Sarcoma	unknown	unknown	low (2)	none	none				BRCA2<br>CHEK2<br>CSF1R<br>GTSE1<br>IRF4<br>MYST3<br>SMO
Sarcoma	0%	stable	intermediate (6)	EGFR duplication exons 18-25<br>PIK3CA G1049R - subclonal<br>ARID1A M923fs*13	EGFR:<br>Afatinib<br>Erlotinib<br>Gefitinib<br>Osimertinib<br>Cetuximab<br>Lapatinib<br>Panitumumab<br>7 trials<br>PIK3CA:<br>Temsirolimus<br>Everolimus<br>10 trials				
Sarcoma	30%	stable	low (2)	PDGFRB R561C<br>PAX 5 loss<br>TNFAIP3 A586T	PDGFR:<br>Sunitinib<br>1 trial			8/1/2019	
Sarcoma	0%	stable	low (1)	EGFR duplication exons 18-25<br>CDKN2A/B loss<br>SDHA A437fs*45	Afatinib<br>Dacomitinib<br>Erlotinib<br>Gefitinib<br>Osimertinib<br>8 trials				
Sarcoma	0%	stable	low (2)	none	none			12/1/2020	ARID2<br>ERBB2<br>EXOSC6<br>KMT2C(MLL3)<br>MED12<br>NTRK3<br>RELN<br>S1PR2<br>SPEN<br>TCF3<br>TSC2<br>TSHR
Sarcoma	unknown	stable	low (2)	NOTCH1 A2279V	none				
Sarcoma	0%	stable	low (2)	BCOR-ITD	none				
Sarcoma	5%	stable	low (2)	MYC<br>HMBOX1-NTRK3 fusion	11 trials				

Diagnosis	PD-L1	MS	TMB	Genomic Findings	Recommended Therapies & Trials	Traditional Tx	Targeted Tx	MTB date	VUS
Soft Tissue Fibromatosis	0%	stable	low (0)	none	none				VUS<br>BRIP1<br>GNAS<br>IGF1R<br>JAK2<br>JAK3<br>ROS1
Soft Tissue Fibromatosis	0%	stable	low (4)	APC	none				
Squamous Cell Carcinoma	10%	stable	low (0)	INPP4B	none				AXIN1<br>FANCG<br>FGF19<br>FLT3<br>GSK3B<br>KDR<br>PTPRO<br>VHL
Synovial Sarcoma	0%	stable	low (1)	CDK4<br>SS18-SSX1 fusion<br>MUTYH G382D	Palbociclib<br>Ribociclib<br>10 trials				
Synovial Sarcoma	0%	stable	low (2)	SS18 SSX1 fusion	1 trial				ATRX E1464del<br>BRSK1 P764A<br>EP300 K1542E & Q2268del<br>FAM123B P750S<br>MKI67 T2509M
Synovial Sarcoma	0%	stable	low (2)	SS18-SSX1 fusion	1 trial				
Synovial Sarcoma	30%	stable	low (0)	SS18-SSX2 fusion<br>KMT2C (MLL3)<br>V884fs*29	NCT02638428 (SS18-SSX2)<br>NCT02389309 (SS18-SSX2)				
T-ALL	unknown	stable	unknown	KRAS<br>CDKN2A/B<br>MET<br>U2AF1<br>NOTCH1	KRAS<br>Selumetinib<br>Trametinib<br>1 trial				AURKB<br>FGFR3<br>KEAP1<br>KMT2A (MLL)<br>MAP3K1<br>NTSC2<br>PCLO<br>SMARCA4<br>TSC1<br>WDR90
Teratoma	unknown	stable	low (1)	U2AF1 R156H	none				BIRC3<br>BRCA1<br>CDH1<br>ICK<br>MLL2<br>MTOR<br>MYST3<br>PCLO<br>PLCG2<br>S1PR2<br>SOX10<br>STK11<br>TSC1
Wilms	0%	stable	low (1)	CTNNB1 S45Y	10 trials	DD4A<br>EOT 6/26/2020			ALK<br>CASP8<br>FUBP1<br>KMT2A (MLL)<br>RBM10<br>RNF43<br>TEK<br>TIPARP<br>TSC1
Wilms	0%	stable	low (1)	FBXW7<br>SPOP<br>TP53	FBXW7<br>Everolimus<br>Temsirolimus			9/6/2021	CTNNB1<br>PCLO<br>LRP1B<br>ZNF703<br>MAP3K6<br>NOTCH1
Wilms	unknown	stable	low (4)	FAM123B R353*<br>TP53 C176_P177insC	none				
Wilms	0%	stable	low (2)	BRCA1	Niraparib<br>Olaparib<br>Rucaparib<br>Talazoparib<br>10 trials			3/21/2021	
Wilms	0%	stable	low (0)	POLE R1634H	none				
